# Tinnitus is associated with reduced sound level tolerance in adolescents with normal audiograms and otoacoustic emissions

**DOI:** 10.1038/srep27109

**Published:** 2016-06-06

**Authors:** Tanit Ganz Sanchez, Fernanda Moraes, Juliana Casseb, Jaci Cota, Katya Freire, Larry E. Roberts

**Affiliations:** 1 University of São Paulo School of Medicine, São Paulo, Brazil; 2 Instituto Ganz Sanchez, São Paulo, Brazil; 3 Association of Interdisciplinary Research and Divulgation of Tinnitus, São Paulo, Brazil; 4 Department of Psychology Neuroscience and Behaviour, McMaster University, Hamilton, Ontario, Canada

## Abstract

Recent neuroscience research suggests that tinnitus may reflect synaptic loss in the cochlea that does not express in the audiogram but leads to neural changes in auditory pathways that reduce sound level tolerance (SLT). Adolescents (N = 170) completed a questionnaire addressing their prior experience with tinnitus, potentially risky listening habits, and sensitivity to ordinary sounds, followed by psychoacoustic measurements in a sound booth. Among all adolescents 54.7% reported by questionnaire that they had previously experienced tinnitus, while 28.8% heard tinnitus in the booth. Psychoacoustic properties of tinnitus measured in the sound booth corresponded with those of chronic adult tinnitus sufferers. Neither hearing thresholds (≤15 dB HL to 16 kHz) nor otoacoustic emissions discriminated between adolescents reporting or not reporting tinnitus in the sound booth, but loudness discomfort levels (a psychoacoustic measure of SLT) did so, averaging 11.3 dB lower in adolescents experiencing tinnitus in the acoustic chamber. Although risky listening habits were near universal, the teenagers experiencing tinnitus and reduced SLT tended to be more protective of their hearing. Tinnitus and reduced SLT could be early indications of a vulnerability to hidden synaptic injury that is prevalent among adolescents and expressed following exposure to high level environmental sounds.

Tinnitus (chronic ringing of the ears) reduces quality of life for millions worldwide. Although the majority of cases are diagnosed after the age of 50 years, persistent tinnitus can occur at any age including in childhood and adolescence^1^. Neuroscience research suggests that most cases of tinnitus are caused, not by pathological increased auditory nerve activity in the cochlea related to otological disease, but by neuroplastic changes that occur in central auditory pathways following deafferentation of those pathways by damage to the cochlear transduction mechanism (inner and outer hair cells and their stereocilia) or to auditory nerve fibers (ANFs) that convey sound information from ribbon synapses on inner hair cells to the brain^2^,^3^,^4^,^5^. Damage to these cochlear structures (sensorineural hearing loss) can increase thresholds for sound detection measured by the clinical audiogram, and accordingly, audiometric hearing loss putatively caused by noise exposure or the aging process is present in most tinnitus sufferers^6^.

However, not all tinnitus patients have abnormal audiograms. Cochlear pathology in these cases may involve, not low-threshold ANFs that are tapped by the audiogram, but high-threshold ANFs that depolarize (fire) only to suprathreshold sounds. Studies in a mouse model of hearing loss found that ~45% of ribbon synapses on high-threshold ANFs were permanently lost following exposure to a level of sound similar to that of some discotheques, with no loss of ribbon synapses on low threshold ANFs or persisting structural damage to cochlear hair cells or their stereocilia^7^,^8^,^9^,^10^. Consequently, wave I of the auditory brainstem response (ABR; wave I measuring neural output from the cochlea) was reduced in exposed compared to unexposed animals when the ABR was evoked by supra-threshold but not by threshold-level sounds. Similar results have been observed in tinnitus patients with normal audiograms; ABR wave I was reduced in patients compared to controls when evoked by noise clicks presented at ≥80 dB SPL but not at lower intensities^11^,^12^. Notably, the amplitude of the later-occurring ABR wave V (generated by neural sources in the auditory midbrain) was either normal^11^ or increased^12^ in the tinnitus patients, suggesting that neural gain had increased in the midbrain to compensate for reduce output from the cochlea. Increased central gain following hidden synaptic loss may explain why the perceived loudness of sounds grows faster with physical sound intensity in tinnitus patients than in controls (sound intolerance) when audiometric hearing loss is absent^13^. Synaptic ribbon loss induced by noise exposure in a mouse model is similarly known to increase behavioral responses to suprathreshold sound^9^. These findings suggest that tinnitus and reduced sound level tolerance (SLT) in humans could reflect synaptic damage to high-threshold ANFs that is not detected by the audiogram (“hidden hearing loss”). Notably, such losses induced by noise trauma in young animals are known to accelerate the pace of progressive age-related changes in cochlear physiology that are otherwise expressed much later in life^14^.

These observations call for study of the prevalence of tinnitus and reduced SLT among adolescents, which could forecast increased risk for hearing difficulties in later years. Although the prevalence of tinnitus among children and adolescents estimated from admissions to otolaryngology clinics is low^15^, prevalence studies of nonclinical populations have disclosed rates as discrepant as 6% to 53% in children^16^,^17^,^18^,^19^,^20^,^21^,^22^,^23^ and from 3.5–69% in adolescents^24^,^25^,^26^,^27^,^28^,^29^,^30^. This variability may relate to several factors including a reliance on questionnaires for tinnitus assessment, the particular youthful populations that were studied, and their prior experience with environmental sounds that may be pathogenic for tinnitus and/or hidden hearing loss. We therefore sought to estimate the prevalence of tinnitus and of reduced SLT among teenagers by questionnaire and by psychoacoustic methods. The results were related to audiometry measured to 16 kHz, otoacoustic emissions measured at several frequencies (these emissions assessing the integrity of the cochlear transduction mechanism), and potentially risky leisure listening habits assessed by questionnaire.

## Methods

### Design and setting

The study was conducted in a well-known private school in Sao Paulo, Brazil, after discussions with the director and teachers so as to protect the students and avoid interference with scholarly activities. Ethical approval was obtained by the University of São Paulo Medical School in accordance with the Declaration of Helsinki and the whole methodology was carried out in accordance with the approved guidelines. All data were collected by the same professional team (2 otologists and 2 audiologists).

### Sample selection

Participants were recruited through an email sent to the parents of all teenagers of the school which explained the research, requested parental signed informed consent, and assured parents that procedures would be performed inside the school but outside the class schedule. Of the 470 families contacted 207 agreed to participate, and from them 170 teenagers volunteered bringing the informed consent signed by parents and composing the final sample. No specific exclusion criteria were applied, but students with wax in the ear canal or ear/nose/throat infections had their evaluation postponed to a day when these issues had cleared. All participants were Caucasians. Their age ranged from 11–17 years old (mean 14.1 years, SD = 2), and 61.1% were males.

### Procedures

Two procedures were performed. The first consisted of otoscopy and a detailed questionnaire administered by an otolaryngologist (Supplementary Table 1s). The questionnaire addressed the student’s self-perception of tinnitus (loudness, duration, and frequency of occurrence), their loudness perception of common daily sounds, and their detailed listening habits including the extent to which they were exposed to noisy environments. Subjects reporting tinnitus were subsequently asked to rate its loudness and annoyance on separate visual analog scales ranging from 0–10 (low to maximum loudness/annoyance).

The second procedure was an assessment of hearing function administered by an audiologist inside an acoustic chamber (Redusom, São Paulo). The following psychoacoustic measurements were taken: (1) Hearing thresholds by the 2-pulse method were measured at 14 frequencies (0.25–16 kHz) using a two-channel Ziptom clinical audiometer (Sancout) with Sennheiser HDA200 earphones (Wedemark). (2) Loudness discomfort levels (LDL, a measure of SLT) were determined for tones of 0.5, 1, 2, and 4 kHz, using the same equipment. Sound pressure level was increased gradually at each frequency until discomfort was reported, at which time the measurement was stopped and sound level in dB was recorded. (3) Transient otoacoustic emissions (TEOAEs) were measured at 1.5, 2, 2.5, 3, 3.5, 4 kHz, and distortion product otoacoustic emissions (DPOAEs) at 2, 4, 6, 8, 10, 12 kHz (TEOAEs and DPOAEs on average 7–10 dB or 10–22 dB above the noise floor, respectively, depending on test frequency). The equipment (OtoRead, Interacoustics) automatically monitored noise level, stimulus stability, and proper placement of the probe. When any aspect was inadequate, the olive was repositioned or replaced and the evaluation restarted.

Before starting the psychoacoustic measurements, all subjects were asked whether they were experiencing tinnitus within the acoustic chamber at that particular moment. If so, psychoacoustic measurement of tinnitus was undertaken. The dominant pitch of tinnitus was determined by asking the teenagers to select from among the sounds used for threshold measurement the frequency that most closely resembled their tinnitus. The level of this sound was then adjusted until it equaled the loudness of their tinnitus in each ear.

### Statistical Analyses

Analyses of variance (ANOVAs) were applied to interval and ratio data using Statistica Version 7.0. Significance levels were adjusted (Greenhouse-Geisser) for repeated measures having more than two levels. Nonparametric 2 × 2 Fisher Exact Tests (two-tailed) were used to assess the questionnaire data.

## Results

### Tinnitus reported by questionnaire was highly prevalent

In order to determine the prevalence of tinnitus by questionnaire, our adolescents were asked “Do you have or have had tinnitus (ringing in the ears) in the last 12 months, except for once/twice and for less than five minutes?” In reply 93/170 teenagers (54.7%) answered “yes”. We identified this group of teenagers as Group GPA. The remaining students (77/170 teenagers; 45.3%) answered “no” and were designated as Group GPB. These two groups correspond to the two columns in Table 1.

We investigated further the characteristics of the tinnitus described by the 93 students in GPA (Table 1s). Fifty-one students (51/93, or 54.8%) reported perceiving their tinnitus after listening to loud music. Nineteen students (19/93, 20.5%) heard their tinnitus at bedtime while twenty-two (22/93, 23.7%) perceived tinnitus any time in silence. Twenty-three students (23/93, 24.7%) reported that tinnitus disrupted their concentration while sixteen (16/93, 17.1%) said it interfered with sleep. Ratings of tinnitus loudness averaged 4.31 (moderate loudness, SD = 1.57, range 1–8) and annoyance 3.57 (low to moderate annoyance, SD = 2.12, range 1–10). However, nine subjects in GPA (9.7% of the sample) gave ratings between 7 and 10, indicating greater annoyance.

### Psychoacoustic assessment corroborated tinnitus and reduced sound level tolerance

During the psychoacoustic measurements, 28.8% of subjects in the total sample (49/170) perceived their tinnitus in the acoustic booth. Almost all of these subjects (93.9%, 46/49) were in GPA. The remaining subjects in GPA (47/93, 50.5%) were not able to detect tinnitus in the chamber, which may have reflected that their tinnitus was sporadic. Thus, it was possible to divide GPA into two subgroups: one consisting of adolescents reporting tinnitus on the questionnaire and hearing it inside the booth (GPA + TB, N = 46), and the second consisting of adolescents reporting tinnitus on the questionnaire but not experiencing it inside the booth (GPA − TB, N = 47). A third subgroup consisted of adolescents in GPB not reporting tinnitus on the questionnaire nor hearing it in the booth (74/77 adolescents, or 96.1% of subjects in GPB). The remaining 3 subjects in GBP (3.9%, 3/77) were aware of tinnitus in the booth for the first time (GPB + TB). These four subgroups are identified within the four cells of Table 1.

**Figure 1:**
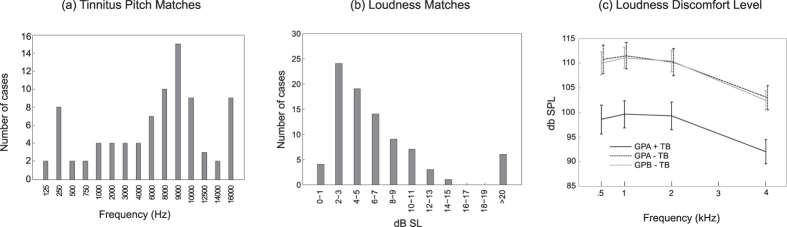
Psychoacoustic measurements of tinnitus and SLT. (**a**) Pitch and (**b**) loudness matches given by adolescents reporting tinnitus in the acoustic chamber. (**c**) Loudness Discomfort Levels reported by adolescents reporting tinnitus on the questionnaire and experiencing it in the acoustic booth (GPA + TB), adolescents reporting tinnitus on the questionnaire but not in the booth (GPA − TB), and adolescents reporting no tinnitus on both measures (GPB − TB). Error bars are ±1 SE.

Psychoacoustic assessments of tinnitus were subsequently conducted for the 49 students who experienced tinnitus in the booth (GPA + TB = 46 and GPB + TB = 3). Tinnitus was bilateral in 38/49 subjects, and unilateral in 11/49 (7/11 left-sided), giving a total of 87 ears for pitch and loudness matching. The majority of pitch matches (Fig. 1a) were located between 6 and 10 kHz (mean pitch = 7073.5 Hz, SD = 4929 Hz). Loudness matches (Fig. 1b) taken at the frequencies chosen for pitch matching averaged 6.74 dB SL (SD = 5.67 dB, range 1–29 dB) with 74.7% (65/87 ears) falling between 2 and 9 dB SL (Fig. 1b). A majority of teenagers reporting tinnitus in the booth (30/49, 61.2%) ascribed a tonal quality to their tinnitus while the remaining subjects likened it to a band of noise. There was no significant effect of the side of tinnitus in bilateral cases on either of these measures.

LDLs were measured for all subjects. Figure 1c reports LDLs for GPA + TB, GPA − TB, and GBP-TB, groups for which the sample size was sufficient for meaningful comparisons. Subjects in GPA + TB expressed loudness discomfort at sound pressure levels that were on average 11.3 dB lower than those of the remaining subgroups, which did not differ from one another (main effect of group F(2,164) = 13.2, p < 0.00001). LDLs decreased at 4 kHz compared to lower frequencies (p < 0.00001), equally in all groups. These findings were not altered when subjects who reported having been exposed to loud sounds within the last 14 hours (11.2% or 19/170 subjects, item #6 in Table 2 later) were excluded from the analysis. With these subjects removed LDL was on average 11.6 dB HL lower in Group GPA + TB compared to the other groups (p < 0.00003) which did not differ from one another.

### Audiometry and otoacoustic emissions did not identify teenagers reporting tinnitus

**Figure 2:**
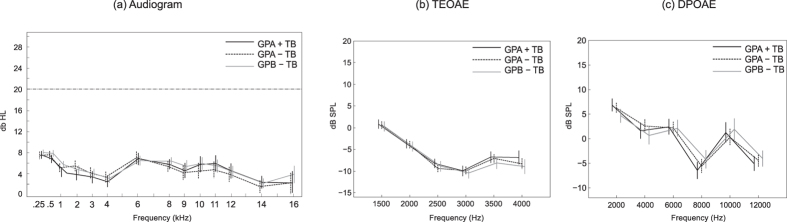
Hearing thresholds and otoacoustic emissions measured in groups GPA + TB, GPA − TB, and GPB − TB. (**a**) Audiogram measured to 16 kHz (20 dBHL is indicated by the broken line). (**b**) Transient and (**c**) distortion product otoacoustic emissions. Error bars are ±1 SE.

Hearing thresholds, TEOAEs, and DPOAEs are reported in Fig. 2a–c respectively, for subgroups GPA + TB, GPA − TB, and GPB − TB. Hearing thresholds (Fig. 2a) were 15 dB HL or better in 97.6% of ears over all frequencies (0.25 to 16 kHz) for all subjects in the experiment. A main effect of frequency was found on hearing thresholds (p < 0.00001), which reflected a small upward shift between 4 and 6 kHz that occurred in each subgroup. Thresholds did not differ between the ears at any frequency in bilateral tinnitus; however, when averaged over all frequencies thresholds were 2.14 dB HL higher in the left compared to the right ear for unilateral left ear tinnitus, and 1.43 dB HL higher in the right compared to the left ear for unilateral right ear tinnitus. These small differences were significant when contrasted to bilateral cases (F (2,45) = 4.16; p = 0.021), owing mainly to thresholds averaging 10 dB HL above 9 kHz in the left ear only, in left ear tinnitus. Importantly, Fig. 2a shows a high degree of overlap among the subgroups in hearing thresholds at each frequency, such that no group differences were found at any frequency.

Similar results were obtained for TEOAEs and DPOAEs. Main effects of frequency were found for both measures (p < 0.00001 in each case), but in no case did main effects or interactions involving subgroup approach significance. TEOAEs were reduced for frequencies above 2 kHz compared to lower frequencies (p < 0.0001), and more so in the left ear (p = 0.031). DPOAEs were lowest at 8 and 12 kHz (p < 0.0001), and were again somewhat lower in the left ear overall (p = 0.027). Most important, there was high overlap among the subgroups at each frequency in TEOAEs and DPAOEs, such that neither measure discriminated between subjects reporting tinnitus in the sound booth (GPA + TB) or only on the questionnaire (GPA − TB) and subjects not reporting tinnitus in either assessment (GPB − TB).

In a further analysis, audiometric thresholds were averaged between 6–16 kHz (the region where most tinnitus pitches were focused) for each subject, and those subjects occupying the bottom 10% and top 10% of the distribution (n = 170) were identified. If elevated thresholds are associated with tinnitus, subjects in the top 10% of this distribution should come predominantly from groups GPA + TB and GPB + TB, whereas subjects not reporting tinnitus in the booth (GPA − TB) or no tinnitus at all (GPB − TB) should dominate in the bottom 10% of the distribution. However, subjects in the bottom 10% and top 10% were distributed randomly among all subgroups, such that no pair-wise contrast among the subgroups was significant (minimum p = 0.17, contrasting GPA + TB + GPA − TB with GPB − TB, one-tail Fisher exact tests). Similar results were obtained for TEOAEs and DPOAEs averaged over all frequencies. Thus, while small differences were detected between the ears in these measures particularly for unilateral tinnitus, we could not distinguish between the presence or absence of tinnitus among any of our cohorts on the basis of audiometric or otoacoustic data.

The same analysis was performed for LDLs, collapsing all subjects into a single distribution. In this case, 23.9% of adolescents in GPA + TB were in the lower tail (low LDL/SLT) of the distribution and 4.3% in the upper tail, compared to 2.1% and 12.8% respectively for subjects in GPA − TB (p < 0.000001, Fisher 2-tail test). A contrast of GPA − TB and GPB − TB on this measure was not significant. Thus, while neither the audiogram nor otoacoustic emissions could identify subjects reporting tinnitus in the sound booth compared to the other groups at a probability exceeding chance, LDL did so with a high level of significance.

### Potentially risky leisure habits were near universal

Reports of risky listening habits are summarized in Table 2. Adolescents in GPA + TB reported near universal listening to music with ear buds (Q4, 95.6% of 46 subjects), attending parties, shows, raves with loud sounds (Q5, 89.1% of 46 subjects), and near universal use of mobile phones excluding use for texting and internet searches (Q7, 95.6% of 46 subjects). Similar exposures were reported by GPA − TB and GPB − TB, such that no significant differences were found among the subgroups in affirmative answers to these three questions.

**Figure 3:**
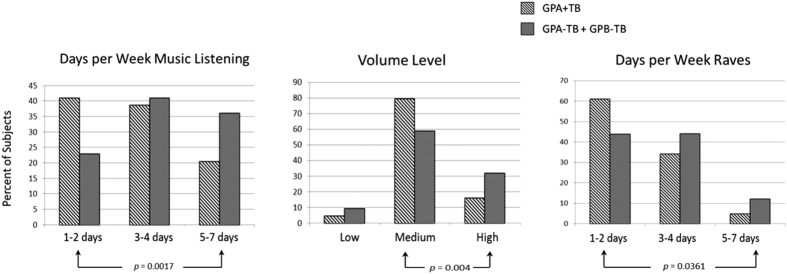
Reports of potentially risky listening habits. Adolescents experiencing tinnitus during audiometric assessment in a sound booth (GPA + TB) reported listening to music fewer days per week (left panel) and at lower volume levels (middle panel), and spending fewer days per week attending parties, shows, and raves where loud sounds were present (right panel), compared to adolescents not reporting tinnitus (GPA − TB + GPB − TB). P-values denote significant interactions between group and level (two-tail Fisher exact tests).

However, evidence for statistically significant differences among the subgroups was found when the extent of sound exposure was examined in more detail. Figure 3 contrasts subjects with tinnitus in the booth (GPA + TB) and those without tinnitus (GPA − TB + GPB-TB) for their replies to questions about the number of days per week on which they listened to music via ear phones (Q4), volume levels when listening to music (Q4), and their attendance at events with loud sounds (Q5). Subjects belonging to GPA + TB reported listening to music via ear buds fewer days each week (1–2 days versus 5–7 days, p = 0.0017) and listening at lower volume levels (medium versus high, p = 0.004) compared to their counterparts without tinnitus (2 × 2 Fisher Exact Tests). They also reported attending fewer parties, shows, and raves where loud music was played compared to adolescents not experiencing tinnitus (1–2 days versus 5–7 days, p = 0.0361, 2 × 2 Fisher Exact Test). A larger percentage of adolescents in GPA + TB reported that sounds that did not bother other people did bother them (Q3, 69.6% of 46 subjects, p = 0.0037), and that they experienced tinnitus after attending parties, shows or raves (Q5, 73.2% of 46 subjects, p = 0.0005) more frequently than did adolescents in the other two groups collapsed (Table 2).

## Discussion

More than half of the 170 adolescents examined in this study (54.7%) reported prior experience of tinnitus when assessed by questionnaire, while 28.8% of the total sample experienced tinnitus when a psychoacoustic examination was subsequently conducted inside an acoustic chamber. Of the latter subjects (GPA + TB), 93.9% had also reported a history of tinnitus on the questionnaire, suggesting a persisting percept in these cases. The tinnitus pitch and loudness matches provided by these subjects were congruent with those typical of adult chronic tinnitus sufferers tested in a similar acoustic environment^6^,^31^. Hearing thresholds measured to 16 kHz and otoacoustic emissions measured up to 12 kHz were clinically normal in all adolescents and did not discriminate between those reporting or not reporting tinnitus in the acoustic booth. However, LDLs were on average 11.3 dB lower for adolescents experiencing tinnitus in the acoustic chamber compared to other adolescents. Thus, LDL was the single psychoacoustic examination that distinguished adolescents with or without tinnitus during audiological assessment.

Although reduced SLT was reported by adolescents with verified tinnitus, its source remains to be determined. Reduced SLT could be a consequence of extracochlear factors such as apprehension evoked by moderately loud sounds when tinnitus has been experienced, increased auditory attention which has been reported in tinnitus sufferers^32^,^33^,^34^, or increased stress related to the experience of tinnitus or its predisposing lifestyle factors^35^. In one large epidemiological survey of adults with tinnitus^35^, “emotional exhaustion” (defined as the experience of “feeling drained of energy due to prolonged stress”) was a strong predictor of tinnitus and reduced SLT. Stimulation of glucocorticoid receptors by dexamethasone (a synthetic stress hormone) also reduced SLT in individuals with tinnitus^36^, although the magnitude of this effect (LDL reduced by 3 dB) was small compared to the decrease of 11.3 dB seen in our adolescents in GPA + TB. Alternatively, hidden hearing loss could be a mechanism underlying reduced SLT and tinnitus. Moderate noise trauma in an animal model is known to cause a permanent loss of almost half of ribbon synapses on high threshold ANFs and to reduce wave I of the ABR evoked by suprathreshold but not by low level sounds^7^. Such losses are followed by compensatory changes in central auditory pathways that increase behavioral responses to sound stimuli^9^. Similarly, wave I of the ABR is reduced in adult tinnitus patients with normal audiograms^11^,^12^, while ABR wave V^12^ and the perceived loudness of sounds^13^ are increased in such patients. Increased neural responses to suprathreshold sounds may reflect a loss of inhibition in central auditory pathways which is known to occur after cochlear hearing loss^3^,^37^ or neuroplastic mechanisms that increase the input-output functions of deafferented neurons so as to preserve their operation within a prescribed dynamic range^38^,^39^,^40^. Either of these mechanisms operating after hidden hearing loss may underlie reduced SLT observed in adult tinnitus patients with normal audiograms and in adolescents in GPA + TB.

Because noise exposure is an established risk factor for tinnitus, one might expect that adolescents experiencing tinnitus and reduced SLT would have reported listening habits that exposed them more often or to higher levels of environmental sound than other adolescents. However, analysis of listening habits by questionnaire did not support this hypothesis. When risky listening practices (which were near universal in our adolescents) were examined in more detail, subjects in GPA + TB reported listening to music by ear buds somewhat less often and at lower volume levels than did subjects not experiencing tinnitus in the audiometric chamber. They also reported attending fewer sound-exposing events per week, and to experience tinnitus more often after such events, than did subjects with no history of tinnitus. These differences, while statistically significant, need to be interpreted with caution, given the retrospective nature of such reports and the possibility that memory may be biased by current perceptual experience. Furthermore, we cannot discount that adolescents in GPA + TB may have been exposed to higher levels of sound exposure than other adolescents in their more distant histories. Notwithstanding these limitations, our results allow the possibility that adolescents in GPA + TB may have experienced tinnitus and reduced SLT, not because they were exposed to more or higher levels of sounds, but because they were primarily more sensitive to the sounds they heard. Consistent with this hypothesis, adolescents in GPA + TB were more likely to report on their questionnaires that sounds that did not bother other people did bother them, which is compatible with clinical hyperacusis, and to experience tinnitus more often after attending parties shows or raves, compared to the other two groups collapsed (Table 2). These results could reflect a vulnerability to the deleterious effects of environmental sound exposure that is expressed as tinnitus and reduced SLT when sound exposure levels are sufficient, which may have been the case in our sample. Impairments of temporal processing have been reported among young listeners with normal audiograms that appear to reflect a similar vulnerability unmasked by a history of sound exposure^41^.

The hypothesis that many adolescents may be vulnerable to hidden hearing impairment has implications for hearing abilities later in life. Synaptic loss induced in mature young animals by a single noise trauma (100 dB SPL for 2 hours) has been shown to accelerate the pace of age-related losses of spiral ganglion neurons, synaptic ribbons, and cochlear hair cells that otherwise occur gradually and to a lesser degree over the life span in unexposed controls^7^,^14^. Similarly, age-related audiometric hearing loss in men correlates with their histories of noise exposure^42^. The high degree of sound exposure and prevalence of tinnitus (54.5% by questionnaire, 28.8% by psychoacoustic assessment) that we observed among our adolescents is a cause for concern, given these findings. The prevalence of tinnitus assessed by questionnaires has returned estimates of 46.9% of 435 students between 10–12 years old^29^, of 36.8% of 428 adolescents between 11–18 years old^24^, and in 38.4% of 125 students with a mean age of 16.7 years^27^. Our finding of 54.7% of adolescents reporting tinnitus by questionnaire was not remarkably higher than these values. A single notable exception is a prevalence of 3.5% obtained from 8179 female adolescents belonging to low socioeconomic status^26^. Considering that we studied male and female Caucasian teenagers from medium to upper socioeconomic status enrolled in a private school, such differences (54.7% vs 3.5%) may relate to the availability of sound listening devices and affordable conditions enabling leisure habits that teenagers want to have. Exposure to high levels of recreational sound appears to be increasing among children and adolescents^24^,^27^,^43^,^44^,^45^,^46^,^47^ and is a likely factor contributing to an increasing incidence of hearing loss which has been reported in western industrialized countries^48^,^49^. Consistent with this account, among adolescents of low socioeconomic status regular use of earphones increased from 18.3% in 2001 to 76.4% in 2008, and out of the 286 teens who reported tinnitus in this study, 99.7% were daily users of ear phones^26^.

The prevalence of tinnitus confirmed by audiometric assessment in 28.8% of our adolescents poses a challenge for future hearing health. The listening habits described by our adolescents may be typical of those in many modern societies^47^. It should be noted, however, that not all sound exposures in animal models necessarily lead to synaptic loss^14^. It is also not uncommon for tinnitus diagnosed at an early age to subside over time, only to reappear later in life as a result of changes in cochlear function related to the cumulative effects of sound exposure or to age-related changes in central aspects of hearing^50^,^51^,^52^. The tinnitus experienced by adolescents in GPA + TB may similarly subside over time, returning later to follow a similar course. Longitudinal studies involving cohorts such as those examined here will be needed to evaluate risks to future hearing health posed by tinnitus and reduced SLT experienced in adolescence. Assessment of hidden hearing loss as a possible contributor calls for studies employing electrophysiological measures such as wave I of the ABR or envelope following responses that have been shown to be sensitive to synaptic loss induced in animals models by noise exposure^53^ and to be altered in adult humans with tinnitus and normal audiometric hearing^11^,^12^.

## Additional Information

**How to cite this article**: Sanchez, T. G. *et al*. Tinnitus is associated with reduced sound level tolerance in adolescents with normal audiograms and otoacoustic emissions. *Sci. Rep*. **6**, 27109; doi: 10.1038/srep27109 (2016).

## Supplementary Material

Supplementary Information

## Figures and Tables

**Table 1:** Tinnitus reported on the questionnaire and experienced in the sound booth.

		Tinnitus reported in the questionnaire		Total
		YES (GPA)	NO (GPB)	
**Tinnitus experienced in the sound booth (TB)**	YES	46 (GPA + TB)	3 (GPB + TB)	49
	NO	47 (GPA − TB)	74 (GPB − TB)	121
	TOTAL	93	77	170

GPA: All adolescents who reported tinnitus in the questionnaire.

GPA + TB: Adolescents in GPA who also reported tinnitus in the acoustic booth.

GPA − TB: Adolescents in GPA who did not perceive tinnitus in the acoustic booth.

GPB: All adolescents who did not report tinnitus in the questionnaire.

GPB + TB: Adolescents in GPB who reported tinnitus in the acoustic booth.

GPB − TB: Adolescents in GPB who did not report tinnitus in the acoustic booth.

**Table 2:** Replies to questionnaire.

		GPA + TB	GPA − TB	GPB − TB
		n = 46	n = 47	n = 74
Q1	Hard to hear or understand? (%yes)	30.4	31.9	27.0
Q3	Do sounds bother you? (% yes)	69.6*	53.2	47.3
Q4	Listen to music with ear phones? (%yes)	95.5	89.4	91.9
	Low Volume	4.5†	9.5	8.8
	Medium volume	79.5	50.0	67.6
	High Volume	15.9	40.5	23.5
	One-two days/week	40.9◊	23.8	32.4
	Three-four days/week	38.6	45.8	54.1
	Five-seven days/week	20.5	31.0	60.6
Q5	Attend parties/shows with loud sound? (% yes)	89.1	80.9	78.4
	Tinnitus after?	73.2*	63.2	32.8
Q6	Exposed to loud sound in last 14 hours? (% yes)	21.7*	10.6	8.1
Q7	Use a mobile phone? (%yes)	95.7	95.7	95.9

Entries are percent of subjects reporting. Questions (Q) are given in full text in Table 1s.

^*^GPA + TB *vs* GPA − TB + GPB − TB p = 0.0180 or better. See text for details.

^†^Interaction Low/High Volume × GPA + TB *vs* GPA − TB + GPB − TB p = 0.0044.

^◊^Interaction 1–2/5–7 days × GPA + TB *vs* GPA − TB + GPB − TB p = 0.0017.

## References

[b1] Gilles A., Van Hal G., De Ridder D., Wouters K. & Van de Heyning P. Epidemiology of noise-induced tinnitus and the attitudes and beliefs towards noise and hearing protection in adolescents. PLoS One 8, e70297. doi: 10.1371/journal.pone.0070297 (2013). 23894638 PMC3722160

[b2] Roberts L. E. . Ringing ears: The neuroscience of tinnitus. J. Neurosci. 30, 14980–14986 (2010). 21068300 10.1523/JNEUROSCI.4028-10.2010 PMC3073522

[b3] Kaltenbach J.A. Tinnitus: Models and mechanisms. Hear. Res. 276, 52–60 (2011). 21146597 10.1016/j.heares.2010.12.003 PMC3109239

[b4] Eggermont J. J. The Neuroscience of Tinnitus Oxford University Press, Oxford, UK (2012).

[b5] Noreña A. J. & Farley B. J. Tinnitus-related neural activity: Theories of generation, propagation, and centralization. Hear. Res. 295, 161–171 (2013). 23088832 10.1016/j.heares.2012.09.010

[b6] Roberts L. E., Moffat G., Baumann M., Ward L. M. & Bosnyak D. J. Residual inhibition functions overlap tinnitus spectra and the region of auditory threshold shift. J. Assoc. Res. Otolaryngol. 9, 417–435 (2008). 18712566 10.1007/s10162-008-0136-9 PMC2580805

[b7] Kujawa S. G. & Liberman M. C. Adding insult to injury: cochlear nerve degeneration after “temporary” noise-induced hearing loss. J. Neurosci. 29, 14077–14085 (2009). 19906956 10.1523/JNEUROSCI.2845-09.2009 PMC2812055

[b8] Furman A. C., Kujawa S. G. & Liberman M. C. Noise-induced cochlear neuropathy is selective for fibers with low spontaneous rates. J. Neurophysiol. 110, 577–586 (2013). 23596328 10.1152/jn.00164.2013 PMC3742994

[b9] Hickox A. E. & Liberman M. C. Is noise-induced cochlear neuropathy key to the generation of hyperacusis or tinnitus? J. Neurophysiol. 111, 552–564 (2014). 24198321 10.1152/jn.00184.2013 PMC3921399

[b10] Emmerich E. . Effects of discotheque music on audiometric results and central acoustic evoked neuromagnetic responses. Int. Tinnitus J. 8, 13–9 (2002). 14763230

[b11] Schaette R. & McAlpine D. Tinnitus with a normal audiogram: physiological evidence for hidden hearing loss and computational model. J. Neurosci. 31, 13452–13457 (2011). 21940438 10.1523/JNEUROSCI.2156-11.2011 PMC6623281

[b12] Gu J. W., Herrmann B. S., Levine R. A. & Melcher J. R. Brainstem auditory evoked potentials suggest a role for the ventral cochlear nucleus in tinnitus. J. Assoc. Res. Otolaryngol. 13, 819–833 (2012). 22869301 10.1007/s10162-012-0344-1 PMC3505586

[b13] Hébert S., Fournier P. & Noreña A. J. The auditory sensitivity is increased in tinnitus ears. J. Neurosci. 33, 2356–2364 (2013). 23392665 10.1523/JNEUROSCI.3461-12.2013 PMC6619157

[b14] Fernandez K. A., Jeffers P. W. C., Lall K., Liberman M. C. & Kujawa S. G. Aging after Noise Exposure: Acceleration of Cochlear Synaptopathy in “Recovered” Ears. J. Neurosci. 35, 7509–7520 (2015). 25972177 10.1523/JNEUROSCI.5138-14.2015 PMC4429155

[b15] Baguley D. M., Bartnik G., Kleinjung T., Savastano M. & Hough E. Troublesome tinnitus in childhood and adolescence: Data from expert centres. Intern. J. Ped. Otorhinolaryngol. 77, 248–251 (2013). 10.1016/j.ijporl.2012.11.009 23245492

[b16] Nodar R. H. Tinnitus aurium in school age children: a survey. J. Auditory Research 12, 133–135 (1972).

[b17] Graham J. M. Tinnitus in children with hearing loss. Ciba Found. Symp. 85, 172–192 (1981). 6915834 10.1002/9780470720677.ch10

[b18] Leonard G., Black F. & Schramm J. Tinnitus in children. In, Bluestone C. D., Stool S., Arjona S. , Eds. Pediatric Otolaryngology, Philadelphia: W. Saunders, 271–277 (1983).

[b19] Mills R. P. & Cherry J. R. Subjective tinnitus in children with otological disorders. Int. J. Pediatr. Otorhinolaryngol. 7, 21–27 (1984). 6539313 10.1016/s0165-5876(84)80050-6

[b20] Nodar R. & Lezak M. Paediatric tinnitus: a thesis revisited. J. Laryng. Otol. 9, 234–235 (1984).

[b21] Stouffer J., Tyler R. & Booth J. In Tinnitus 91 – Proceedings of IV International Tinnitus Seminar, Vol.1 (ed Aran J. M.) 255–258 (Kugler, 1991).

[b22] Holgers K. M. Tinnitus in 7-year-old children. Eur. J. Pediatr. 162, 276–278 (2003). 12647204 10.1007/s00431-003-1183-1

[b23] Coelho C. C. B., Sanchez T. G. & Tyler R. S. Tinnitus in children and associated risk factors. Prog. Brain. Res. 166, 179–191 (2007). 17956782 10.1016/S0079-6123(07)66016-6

[b24] Bulbul S. F., Muluk N. B., Cakir E. P. & Tufan E. Subjective tinnitus and hearing problems in adolescents. Int. J. Pediatr. Otorhinolaryngol. 73, 1124–1131 (2009). 19467720 10.1016/j.ijporl.2009.04.018

[b25] Zocoli A. M., Morata T. C., Marques J. M. & Corteletti L. J. Brazilian young adults and noise: attitudes, habits, and audiological characteristics. Int. J. Audiol 48, 692–699 (2009). 19863355 10.1080/14992020902971331

[b26] Berg A. L. & Serpanos Y. C. High frequency hearing sensitivity in adolescent females of a lower socioeconomic status over a period of 24 years (1985-2008). J. Adolesc. Health 48, 203–208 (2011). 21257121 10.1016/j.jadohealth.2010.06.014

[b27] Lacerda A. B. M., Gonçalves C. G. O., Zocoli A. M. F., Diaz C. & Paula K. Hábitos auditivos e comportamento de adolescentes diante das atividades de lazer ruidosas. Rev. CEFAC 13, 322–329 (2011).

[b28] Juul J., Barrenäs M. L. & Holgers K. M. Tinnitus and hearing in 7-year-old children. Arch. Dis. Child. 97, 28–30 (2012). 22100742 10.1136/archdischild-2011-300270

[b29] Kim Y. H. . Tinnitus in children: association with stress and trait anxiety. Laryngoscope 122, 2279–2284 (2012). 22886845 10.1002/lary.23482

[b30] Knobel K. A. B. & Lima M. C. M. P. Os pais conhecem as queixas auditivas de seus filhos? Braz J. Otorhinolaryngol. 78, 27–37 (2012). 23108817 10.5935/1808-8694.20120005 PMC9450749

[b31] Noreña A., Micheyl C., Chéry-Croze S. & Collet L. Psychoacoustic characterization of the tinnitus spectrum: Implications for the underlying mechanisms of tinnitus. Audiol. Neurootol. 7, 358–369 (2002). 12401967 10.1159/000066156

[b32] Cuny C., Noreña A., El Massioui F. & Chéry-Croze S. Reduced attention shift in response to auditory changes in subjects with tinnitus. Audiol. Neurootol, 9, 294–302 (2004). 15319555 10.1159/000080267

[b33] Paul B. T., Bruce I. C., Bosnyak D. J., Thompson D. C. & Roberts L. E. Modulation of electrocortical brain activity by attention in individuals with and without tinnitus. Neural Plasticity, Article ID 127824 doi: 10.1155/2014/127824 (2014). PMC4082949 25024849

[b34] Roberts L. E., Husain F. T. & Eggermont J. J. Role of attention in the generation and modulation of tinnitus. Neuroscience and Biobehavioral Reviews, 37, 1754–1773 (2013). 23876286 10.1016/j.neubiorev.2013.07.007

[b35] Hébert S., Canlon B. & Hasson D. Emotional exhaustion as a predictor of tinnitus. Psychother Psychosom 81, 324–326. doi: 10.1159/00033504. 22854311

[b36] Simoens V. L. & Hébert S. Cortisol suppression and hearing thresholds in tinnitus after low-dose dexamethasone challenge. BMC Ear, Nose and Throat Disorders 12, 4 (2012). 10.1186/1472-6815-12-4 PMC3328238 22449242

[b37] Yang S., Weiner B. D., Zhang L. S., Cho S. J. & Bao S. Homeostatic plasticity drives tinnitus perception in an animal model. Proc. Nat. Acad. Sci. USA 108, 14974–14979 (2011). 21896771 10.1073/pnas.1107998108 PMC3169130

[b38] Turrigiano G. G. & Nelson S. B. Homeostatic plasticity in the developing nervous system. Nat Rev Neurosci 5, 97–107 (2004) 14735113 10.1038/nrn1327

[b39] Pozo K. & Goda Y. Unraveling mechanisms of homeostatic synaptic plasticity. Neuron 66, 337–351 (2010). 20471348 10.1016/j.neuron.2010.04.028 PMC3021747

[b40] Zeng C., Nannapaneni N., Zhou J., Hughes L. F. & Shore S. Cochlear damage changes the distribution of vesicular glutamate transporters associated with auditory and nonauditory inputs to the cochlear nucleus. J Neurosci 29, 4210–4217 (2009). 19339615 10.1523/JNEUROSCI.0208-09.2009 PMC4487620

[b41] Bharadwaj H. M., Masud S., Mehraei G., Verhulst S. & Shinn-Cunningham B. G. Individual differences reveal correlates of hidden hearing deficits. J Neurosci 35, 2161–2172 (2015). 25653371 10.1523/JNEUROSCI.3915-14.2015 PMC4402332

[b42] Gates G. A., Schmid P., Kujawa S. G., Nam B. & D’Agostino R. Longitudinal threshold changes in older men with audiometric notches. Hear. Res. 141, 220–228 (2000). 10713509 10.1016/s0378-5955(99)00223-3

[b43] Mahboubi H., Oliaei S., Kiumehr S., Dwabe S. & Djalilian H. R. The prevalence and characteristics of tinnitus in the youth population of the United States. Laryngoscope 123, 2001–2008 (2013). 23606449 10.1002/lary.24015

[b44] Woodford C. & O’Farrel M. High-frequency loss of hearing in secondary school students: an investigation of possible etiologic factors. Language, Speech, and Hearing Services in Schools 14, 22–28 (1983).

[b45] Chemark G. D. & Peters-McCarthy E. The effectiveness of an educational hearing conservation program for elementary school children. Language, Speech, and Hearing Services in Schools 22, 308–312 (1991).

[b46] Montgomery J. K. & Fujikawa S. Hearing thresholds of students in the second, eighth, and twelfth grades. Language, Speech and Hearing Services in Schools 23, 61–63 (1992).

[b47] Vogel I., Brug J., Van der Ploeg C. P. & Raat H. Discotheques and the risk of hearing loss among youth: risky listening behavior and its psychosocial correlates. Health Educ Res 25, 737–747 (2010). 20338977 10.1093/her/cyq018

[b48] Henderson E., Testa M. A. & Hartnick C. Prevalence of noise-induced hearing-threshold shifts and hearing loss among US youths. Pediatrics, 12, 39–46. doi: 10.1542/peds.2010-0926 (2011). 21187306

[b49] Shargorodsky J., Curhan S. G., Curhan G. C. & Eavey R. Change in prevalence of hearing loss in US adolescents J Am Med Assoc 304, 772–778 (2010). 10.1001/jama.2010.1124 20716740

[b50] Roberts L. E. In Perspectives on Tinnitus (eds Baguley D. & Fagelson M.), Ch 2 13–33 (Plural, 2015).

[b51] Llano D. A., Turner J. & Caspary D. M. Diminished cortical inhibition in an aging mouse model of chronic tinnitus. J. Neurosci. 32, 16141–16148 (2012). 23152598 10.1523/JNEUROSCI.2499-12.2012 PMC3517907

[b52] Viana L. M. . Cochlear neuropathy in human presbycusis: Confocal analysis of hidden hearing loss in post-mortem tissue. Hear. Res. 327, 78–88, doi: 10.1016/j.heares.2015.04.014 (2015). 26002688 PMC4554812

[b53] Shaheen L. A., Valero M. D. & Liberman M. C. Towards a diagnosis of cochlear neuropathy with envelope following responses. J. Assoc Res Otolaryngol doi: 10.1007/s10162-015-0539-3 (2015). PMC4636593 26323349

