# Supplementary material for: Tinnitus is associated with reduced sound level tolerance in adolescents with normal audiograms and otoacoustic emissions
Source: Sci Rep. 2016 Jun 6;6:27109. doi: 10.1038/srep27109 (PMC4893619; doi:10.1038/srep27109)
Supplement: Supplementary Information [file srep27109-s1.pdf]

Tinnitus is associated with reduced sound level tolerance in adolescents with normal audiograms and otoacoustic emissions

*Tanit Ganz Sanchez[1,2,3], Fernanda Moraes[2], Juliana Casseb[2], Jaci Cota[2], Katya Freire[3], & Larry E. Roberts[4]

[1]University of São Paulo School of Medicine, São Paulo, Brazil

[2]Instituto Ganz Sanchez, São Paulo, Brazil

[3]Association of Interdisciplinary Research and Divulgation of Tinnitus, São Paulo, Brazil

[4]Department of Psychology Neuroscience and Behaviour, McMaster University, Hamilton, Ontario, Canada

*Corresponding Author:

Tanit Ganz Sanchez, MD, PhD

Av. Padre Pereira de Andrade, 353

São Paulo, SP, Brazil – CEP: 05469-000 Phone/fax (+5511) 3021-5251

Author emails: tanitsanchez@gmail.com; drafmoraes@gmail.com; jucasseb@yahoo.com.br; jacicota@gmail.com; katya@audicare.com.br; roberts@mcmaster.ca

Table 1s. Summary of questionnaire. Each main question was answered "yes" or "no". If "yes", supplementary questions provided further information[1,2]

1. Is it hard to hear or understand?

2. Have you had tinnitus in the last 12mo, but once/twice/few seconds?

When or where:

A. Only at bedtime, less than 50% of the nights
B. Only at bedtime, more than 50% of the nights
C. Anytime you are in silence (day or night)
D. After loud music, but disappears next day
E. After loud music, and lasts 2-3 days to disappear
F. In other situations: ....

Ear of tinnitus: (Right, left, both)
Tinnitus sound: (ringing, hissing, other)
Tinnitus affects: (nothing, concentration, sleep, social life)

3. Are you bothered with sounds that other people are not?

If yes, which sounds?

A. recess	E. whistle	I. people talking
B. classroom	F. cars/motorcycles	J. games
C. Barking dogs	G. radio/TV	K. Other: ....
D. Phone	H. music	

4. Do you use to hear music with ear phones?

Which volume?	( ) high	( ) medium	( ) low
How many hours per day?	( ) < 1h	( ) 1-2h	( ) > 3h
How many days per week?	( ) 1-2d	( ) 3-4d	( ) 5-7d

5. Do you use to go to parties/baladas/shows/raves with loud sounds?

How long do you stay?	( ) < 1h	( ) 1-2h	( ) > 3h
How many days per week?	( ) 1-2d	( ) 3-4d	( ) > 5d

Do you leave them with temporary tinnitus? (yes/no)

6. Have you been exposed to loud sounds in the last 14h? (yes/no)

7. Do you use a mobile phone (except for texting or internet)?

How long per day?	( ) <10min	( ) 10-30min	( ) >30min
How do you use?	( ) speaker	( ) touching ear	( ) ear phone
Which ear do you prefer?	( ) right	( ) left	( ) both

[1] Subjects reporting tinnitus on Question 2 above rated its loudness and annoyance separately on visual analogue scales ranging from 1 (minimal loudness or annoyance) to 10 (maximum loudness and annoyance).

[2] Questions not listed investigated eating habits, otitis, and temporomandibular joint symptoms.
